# Identification of a copper metabolism‐related gene signature for predicting prognosis and immune response in glioma

**DOI:** 10.1002/cam4.5688

**Published:** 2023-03-01

**Authors:** Ling Li, Wenyuan Leng, Junying Chen, Shaoying Li, Bingxi Lei, Huasong Zhang, Huiying Zhao

**Affiliations:** ^1^ Department of Medical Research Center, Sun Yat‐sen Memorial Hospital Sun Yat‐sen University Guangzhou China; ^2^ Zhongshan School of Medicine Sun Yat‐sen University Guangzhou China; ^3^ Department of Neurosurgery, Sun Yat‐sen Memorial Hospital Sun Yat‐sen University Guangzhou China; ^4^ Department of Otolaryngology, Longgang E.N.T hospital & Shenzhen Key Laboratory of E.N.T Institute of E.N.T Shenzhen China

**Keywords:** bioinformatics, cancer immunity, copper metabolism, diffuse glioma, prognosis

## Abstract

**Background:**

Gliomas are highly refractory intracranial cancers characterized by genetic and transcriptional heterogeneity. However, therapeutic options are limited. In the last years, copper‐induced cell death is becoming a prospective treatment strategy for gliomas and other solid tumors, but copper metabolism‐related genes associated with cancer development remain unclear.

**Methods:**

We first collected gene expression data from The Cancer Genome Atlas (TCGA) to identify significantly differentially expressed copper metabolism‐related genes in gliomas. Using these genes, we performed COX regression and Least Absolute Shrinkage and Selection Operator (LASSO) regression to construct the prognostic model. The prognostic value of the model was further validated by CGGA testing set. Subsequently, functional analyses were carried out, including gene set enrichment analysis (GSEA), immune infiltration analysis, and mutation analysis. Finally, the expression levels of these genes were verified by immunohistochemical analysis.

**Results:**

The prognostic model consisted of 7 genes: CDK1, LOXL2, LOXL3, NFE2L2, SLC31A1, SUMF1 and FDX1. According to this prognosis model, glioma patients could be split into the high‐risk group or low‐risk group, and the low‐risk group showed significantly better prognostic survival (*p* < 0.001). Moreover, the high‐risk group had higher levels of immune cell infiltration, immune checkpoint genes expression, and higher tumor mutational burden (TMB), which indicates that they might benefit more from immunotherapy. Finally, we confirmed the expression level of FDX1, SUMF1, and SLC31A1 protein as significantly different in glioblastoma, lower‐grade glioma, and non‐tumor brain tissues by immunohistochemical analysis, and the high expression of FDX1 and SLC31A1 protein was related to poor survival in glioma patients.

**Conclusions:**

Our study could contribute to the prognosis prediction and decision‐making in patients with gliomas.

## Graphical abstract

A copper metabolism‐related gene signature was identified as a prognostic model for glioma, and the prognostic model has good accuracy in predicting patient outcomes in both TCGA and CGGA cohorts. Besides, the high‐risk group had higher levels of immune cell infiltration, immune checkpoint genes expression, and higher tumor mutational burden, suggesting that they might be more likely to benefit from immunotherapy. Our study can provide a reference for the diagnosis and treatment of patients with glioma.

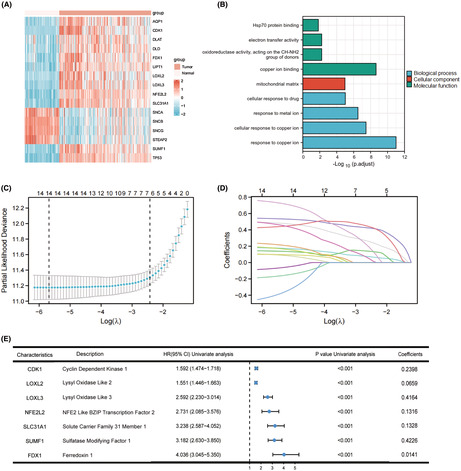

## 1 INTRODUCTION

Gliomas are highly refractory and the most prevalent primary intracranial cancers, comprising 80% of all malignancies in the brain.^1^ Diffuse gliomas consist of lower‐grade gliomas (LGGs), including WHO grade II and III tumors, and glioblastoma (GBM).^2^ The median OS time of patients with diffuse gliomas is 78.1 months for WHO grade II, 37.6 months for WHO grade III, and 14.4 months for WHO grade IV gliomas.^3^ The main treatment of glioma is the combination of surgery and radiotherapy or chemotherapy. However, most of the patients experience limited benefit from them.^4^ Thus, diffuse glioma is still a heavy burden for human health, and new therapies are needed to improve the prognosis of patients.

An important reason hindering the development of novel treatment for gliomas is that gliomas are highly heterogeneous in all molecular subgroups.^5^ Histology alone is insufficient to give the accurate diagnosis and prognostic stratification needed for targeted treatment. Combination of histological assessment with molecular markers facilitates more accurate patient diagnosis and risk stratification. For example, isocitrate dehydrogenase 1 (IDH1) mutation has been identified as a typical favorable prognostic marker.^6^ However, more effective prognostic molecular markers are particularly needed for improving prognosis and long‐term life quality of patients with diffuse gliomas.

In detecting molecular markers related to prognosis of cancers, recent studies have indicated the importance of copper metabolism.^7^ Copper, as an essential cofactor for enzymes and a cellular signaling agent, mediates a range of biochemical processes, including mitochondrial respiration, antioxidation, and hormone and neuropeptide biogenesis. However, excessive copper ion induces cytotoxicity; therefore, intracellular copper is restricted to low concentrations by homeostatic mechanisms to prevent the accumulation of free copper and copper‐induced cytotoxicity.^8^, ^9^, ^10^ Unlike normal tissues, it was proved that tumor cells have a relatively higher concentration of copper, which may contribute to the pathogenesis of it.^11^, ^12^, ^13^, ^14^, ^15^, ^16^, ^17^ Tsvetkov et al. found abnormal elevated copper ions may cause a previously unidentified way of cell death, which induced by aggregation of lipoylated TCA cycle proteins and termed this distinct copper‐dependent cell death mechanism “cuproptosis.”^7^

Copper‐induced cell death is becoming a prospective treatment strategy for glioma and other solid tumors.^18^, ^19^, ^20^, ^21^, ^22^, ^23^, ^24^ Studies showed that copper oxide‐related cytotoxicity can cause severe cell death in C6 glioma cells, which can be prevented by adding copper chelators.^25^ Zheng et al. showed a potential combinatory therapeutic strategy of gliomas, which contains copper.^26^ Moreover, Tsvetkov et al. identified several genes involved in cuproptosis, whose altered expressions are highly correlated across human tumors, making tumors more sensitive to cuproptosis.^7^ Copper metabolism‐related genes thus emerge as promising therapeutic targets and potential prognostic markers for gliomas. However, there are still insufficient studies in constructing prognostic models in diffuse glioma based on copper metabolism, which hindered the clinical application of the copper metabolism‐related genes in diagnosis and therapy design in glioma patients.

In this study, first, we performed a bioinformatic analysis based on mRNA expression and survival data from TCGA, and constructed a prognostic model for glioma patients using copper metabolism‐related genes. Then we validated the model in the CGGA dataset.^27^ Finally, GSEA, immune cell infiltration analysis, and mutation landscape analysis were performed to investigate the underlying mechanisms of the signature in the immune microenvironment and its guiding value for immunotherapy of glioma. Our study could be helpful for the prognosis prediction and decision‐making in patients with gliomas.

## 2 MATERIALS AND METHODS

### 2.1 Collection of copper metabolism‐related genes

Copper metabolism‐related genes were downloaded from the Molecular Signatures Database (MSigDB) version 7.5.1.^28^, ^29^ We also added the previously reported regulatory gene of copper‐induced cell death.^7^ Thus, a set of 111 genes related to copper metabolism was obtained (Table S1).

### 2.2 Datasets and data processing

TCGA dataset and GTEx project was downloaded from UCSC Xena (https://tcga.xenahubs.net), including 529 LGG samples, 168 GBM samples and 263 normal cerebral cortex samples. Clinical data of these patients was acquired from the study of Ceccarelli M et al.^30^ Clinical data and RNA sequencing data of CGGA dataset for 693 patients with glioma (444 LGG samples, 249 GBM samples) were downloaded from CGGA database. (http://www.cgga.org.cn/)^31^ The values of gene expression profiles in these datasets were converted by log_2_ for subsequent analysis.

### 2.3 Differential analysis

Differential analysis was performed between the TCGA glioma samples and normal brain samples by “DESeq2” package in RStudio (version 4.1.3). Differentially expressed genes (DEGs) were filtered by a false discovery rate (FDR) threshold <0.05 and absolute log2‐foldchange>1. From the DEGs, we selected the copper metabolism‐related genes for further analysis.

### 2.4 Building and validating the prognostic model

LASSO regression analysis was applied to construct a prognostic model, which has reduced the overfitting by selecting high‐dimensional prognostic genes using the “glmnet” package. The risk score was calculated using the following formula:

$$\text{Risk Score}=\sum_{i=1}^{n}{\text{expr}}_{\text{genei}}\times {\text{coefficient}}_{\text{genei}}$$

where ${\text{expr}}_{\text{genei}}$ is the normalized gene expression value and ${\text{coefficient}}_{\text{genei}}$ is its coefficient.

Through this formula, each LGG/GBM patient can be calculated with a risk score, and patients were grouped into the high‐risk or low‐risk group according to the median risk score. (Detailed procedures were described in the Supplementary Methods.)

### 2.5 The construction of a nomogram

To better predict the prognosis in both the training and testing cohorts, a nomogram was constructed using the “rms” package. Calibration analysis and ROC curve were used to assess the accuracy of the nomogram. (Detailed procedures were described in the Supplementary Methods.)

### 2.6 GSEA

The “DESeq2” package was used to obtain the DEGs between the high‐ and low‐risk groups in the TCGA set. Then, GSEA^32^ was performed using the “clusterProfiler” package.^33^

### 2.7 Immune cell infiltration analysis

We downloaded the immune infiltration estimation results for GBM/LGG patients in the TCGA database from the Timer2.0 database.^34^

### 2.8 Mutation landscape

Mutational data of patients (*n* = 681) with LGG/GBM from TCGA were obtained from cBioPortal.^35^ The mutational data was analyzed by “maftools” R package^36^ (Supplementary Methods).

### 2.9 Protein expression in HPA database

The Human Protein Atlas (HPA) database (www.proteinatlas.org) was used for comparison of protein expression of prognostic genes between LGG and GBM samples by immunohistochemistry image.^37^

### 2.10 Tissue specimens and patient information

From Department of Neurosurgery, Sun Yat‐sen Memorial hospital, we collected 91 surgical specimens, which had been histopathologically diagnosed gliomas by Department of Pathology of our hospital according to WHO criteria between 2012 and 2022. Among these, 60 surgical specimens with follow‐up survival data were made into tissue microarrays (TMAs), while the rest (*n* = 31) were tissue slides. These samples include 55 GBM specimens, 31 LGG specimens and 5 normal brain tissues (from surgical specimens of patients with cerebrovascular disease) (Supplementary Methods).

### 2.11 Immunohistochemical (IHC) staining

Immunohistochemical analysis was performed using tissue slides or TMAs of human surgical specimens (Supplementary Methods).

## 3 RESULTS

### 3.1 Identification of prognostic copper metabolism‐related DEGs in the TCGA‐LGG/GBM cohort

**FIGURE 1:**
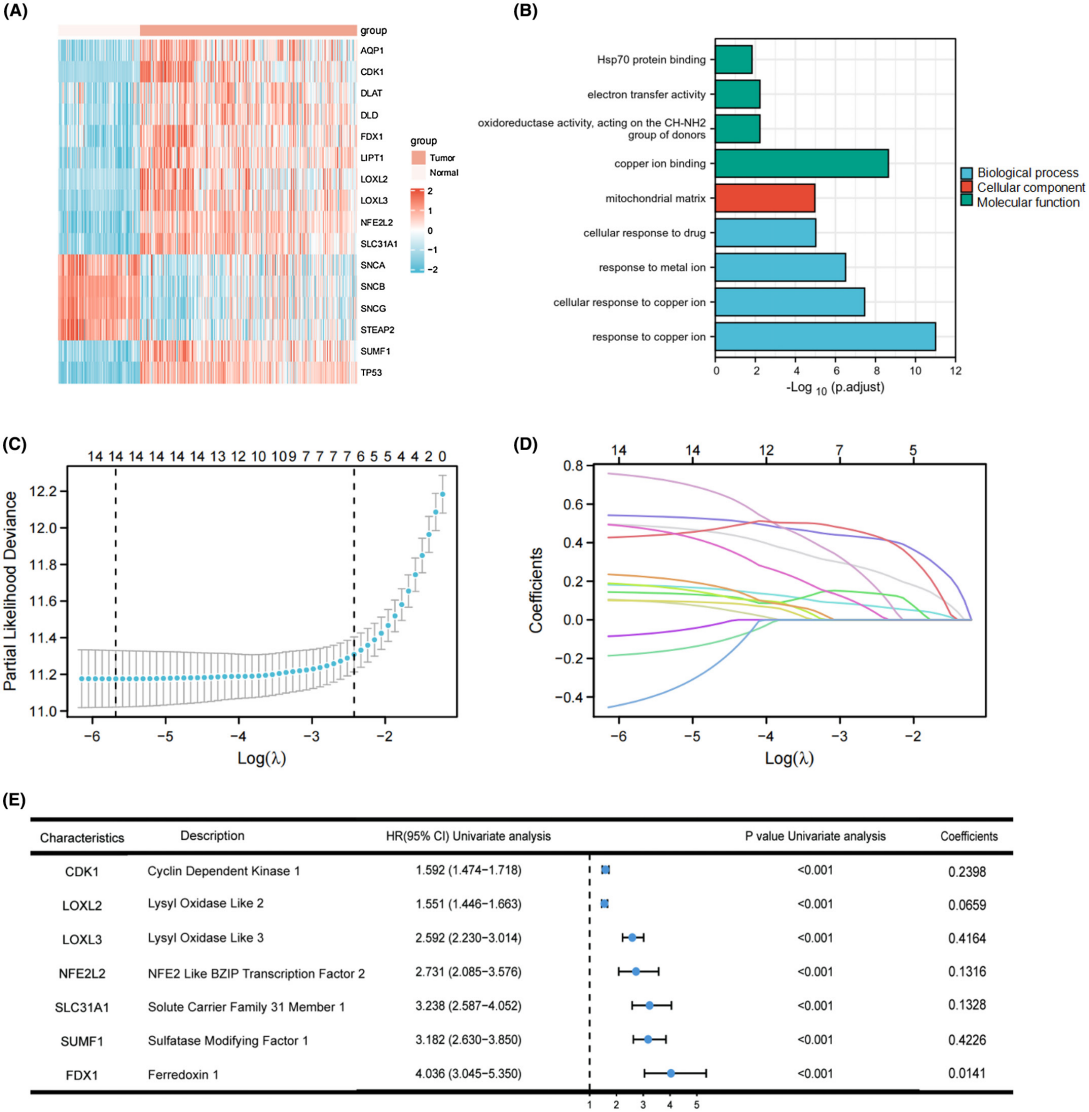
Identification of prognostic copper metabolism‐related DEGs and construction of copper metabolism‐related prognostic model. (A) Heatmap of the expression level of 16 DEGS. (B) Enriched Gene Ontology terms associated with 16 DEGs. (C) By LASSO analysis, seven genes were screened out and λ was 0.0889 at the minimum partial likelihood deviation. (D) Path diagram of lasso regression coefficients. (E) Forest plots showing the results of the univariate Cox regression analysis between gene expression and OS and their coefficients in LASSO analysis.

Figure S1 shows the flowchart of this study. The DEG analysis was performed between TCGA glioma samples and normal brain samples, among them, 39 were identified as copper metabolism‐related DEGs. Then, univariate Cox regression analysis of OS was performed on these DEGs, indicating 34 of them were correlated with OS. From these genes, 18 genes were excluded for further analysis because 8 of them was upregulated in tumor samples with their expression levels positively correlated with survival rate, and 10 genes was downregulated in tumor samples with their expression levels negatively correlated with survival rate. Of the remaining 16 copper metabolism‐related DEGs, 12 were upregulated, and 4 were downregulated in glioma compared to normal tissue expression (Figure 1A). Functional enrichment analyses indicated these genes were significantly (Pcorrection <0.01) enrichment in cellular response to copper ion and copper ion binding (Figure 1B).

### 3.2 Construction and validation of a copper metabolism‐related prognostic model

In order to further refine selected genes mentioned above and construct a prognostic model, we performed LASSO regression analysis of the 15 genes mentioned above (AQP1 was not found in CGGA database, so it was excluded). The results showed that the model had the best fitting effect when the number of genes was 7 and λ was 0.0889 (Figures 1C,D). The results of univariate Cox regression analysis and Lasso regression coefficient of these seven genes are summarized in Figure 1E, which were used for constructing the prognostic model.

**FIGURE 2:**
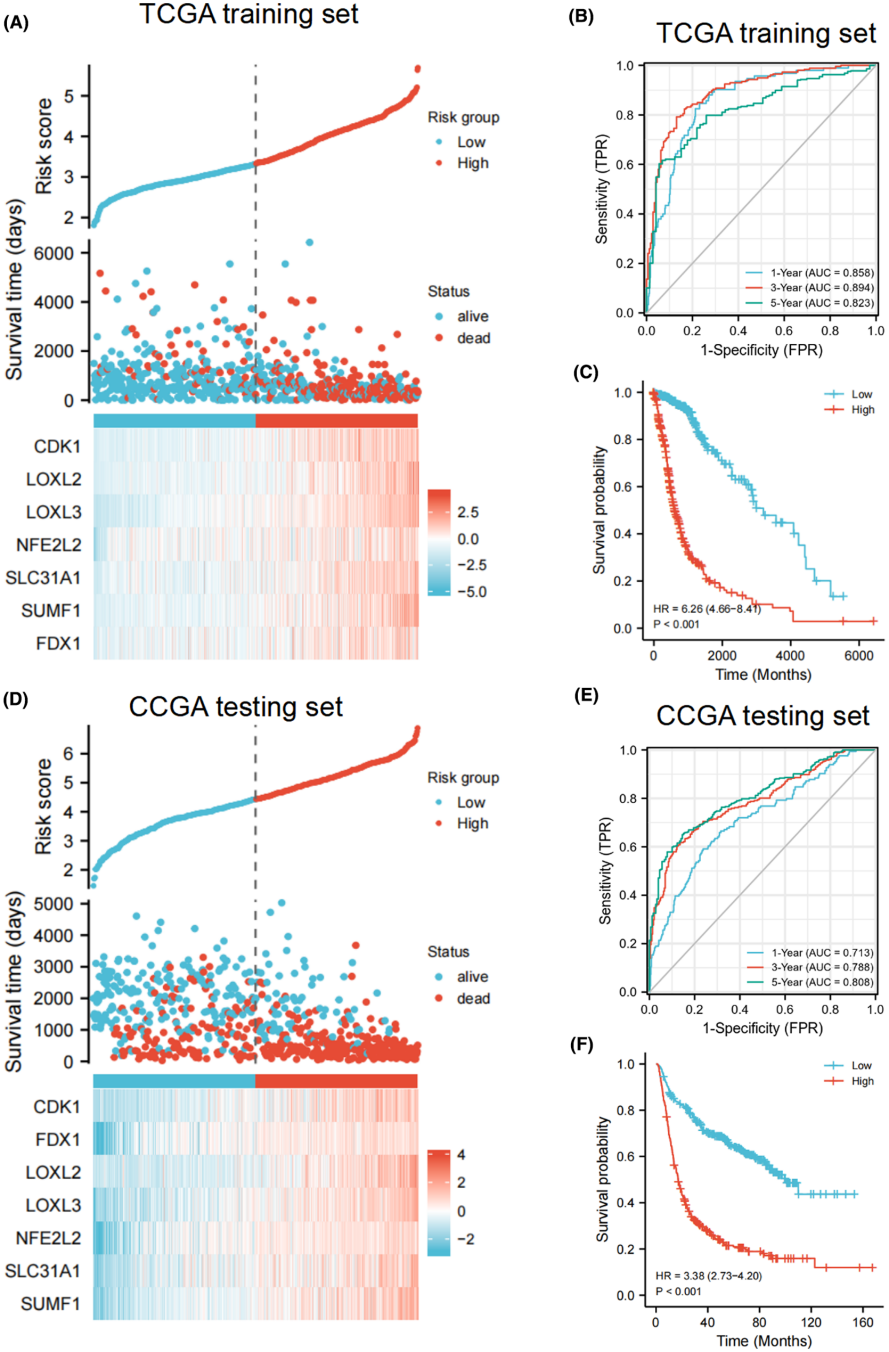
Predictive performance of the prognostic model. Risk score and OS time distributions, and heatmaps of expression levels of the prognostic genes in the (A) training and (D) testing sets. ROC curves of the prognostic model for predicting the 1‐, 3‐, and 5‐year OS times in the (B) training and (E) testing sets. Kaplan–Meier survival analysis was undergone to compare the OS times between the two groups in the (C) training and (F) testing sets.

According to the expression levels and regression coefficients of selected genes, the risk scores were calculated for each cases in both sets. Figure 2A shows the differences between the high‐risk and low‐risk groups in the living state, survival times and the distribution of the prognostic genes expression in TCGA sets, which indicated that patient with a higher risk score may be correlated with a poorer outcome in the TCGA training cohort. All these seven genes displayed higher expression in the high‐risk group than in the low‐risk group. The similar gene expression profiles were observed in the testing set (Figure 2D).

To further evaluate the predictive performance of the gene signature in predicting the prognosis of glioma patients, we performed ROC curve analysis. The results showed that the area under curve (AUC) values of 1‐year reached 0.858, 3‐year reached 0.894, and 5‐year reached 0.823 (Figure 2B), and the OS time of the high‐risk group was significantly shorter than that of the low‐risk group in the TCGA set. (*p* < 0.001, Figure 2C). In the CGGA dataset, we found that the AUC of 1, 3 and 5 years were 0.713, 0.788 and 0.808, respectively (Figure 2E). Similarly, in the CGGA dataset, relative to patients in the low‐risk group, patients in the high‐risk group have a significantly worse prognosis (*p* < 0.001, Figure 2F). These results showed that the gene signature has good performance in predicting the prognosis of patients in both groups.

### 3.3 Clinical stratified analysis

To investigate the correlation between risk score and other clinical characteristics, stratified analysis of risk score was performed according to different clinical characteristics. As shown in Figures S2, in the TCGA set, patients with higher risk score tend to be older than 40 years, to have higher WHO grade, unmethylated MGMT promoter, glioblastoma, non‐codeletion of 1p/19q and wild type IDH1 (all *p* < 0.001). In the CGGA set, stratified analyses of risk score for WHO grade, histological type, 1p/19q codeletion, and IDH1 mutation status were consistent with those in TCGA database (all *p* < 0.001, Figures S2). No significant difference was found in risk score distribution between different age levels or MGMT methylation status (Figures S2). No correlation was observed between risk score and gender in both sets (data not shown). Thus, these findings indicated that risk scores constructed by the seven‐gene signature are able to reflect the clinical status of patients in both TCGA and CGGA sets.

### 3.4 Construction and validation of the nomogram

To further investigate the independence of the risk score as a risk factor for OS time in diffuse glioma patients, univariate and multivariate Cox regression analysis were performed by considering potential risk factors as parameters. As shown in Table 1, in multivariate Cox regression, the risk group was identified as independent risk factor (HR = 2.257, 95% CI = 1.413–3.606, *p* < 0.001). Besides, the WHO grade, age, IDH status, 1p/19q codeletion status were also considered to be independent risk factors related to prognosis, and were used to construct the nomogram.

**TABLE 1:** Univariate and multivariate Cox analysis of OS in TCGA datasets.

Parameters		Univariate Cox analysis		Multivariate Cox analysis	
		HR (95% CI)	*p*‐value	HR (95% CI)	*p*‐value
Age level	Young (≤40)	—	—	—	—
	Old (>40)	4.220 (3.086–5.770)	<0.001	2.809 (1.865–4.233)	<0.001
Gender	Female	—	—	—	—
	Male	1.262 (0.988–1.610)	0.062	—	—
WHO grade	G2	—	—	—	—
	G3	2.999 (2.007–4.480)	<0.001	1.581 (0.999–2.502)	0.051
	G4	18.615 (12.460–27.812)	<0.001	3.567 (2.008–6.335)	<0.001
IDH status	WT	—	—	—	—
	Mutant	0.117 (0.090–0.152)	<0.001	2.171 (1.326–3.554)	0.002
1p/19q codeletion	non‐codel	—	—	—	—
	codel	0.226 (0.147–0.347)	<0.001	0.616 (0.367–1.034)	0.013
MGMT promoter status	Unmethylated	—	—	—	—
	Methylated	0.319 (0.245–0.416)	<0.001	0.833 (0.592–1.171)	0.293
risk level	Low risk	—	—	—	—
	High risk	6.260 (4.660–8.410)	<0.001	2.257 (1.413–3.606)	<0.001

Abbreviations: 95% CI, 95% confidence interval; HR, hazard ratio.

**FIGURE 3:**
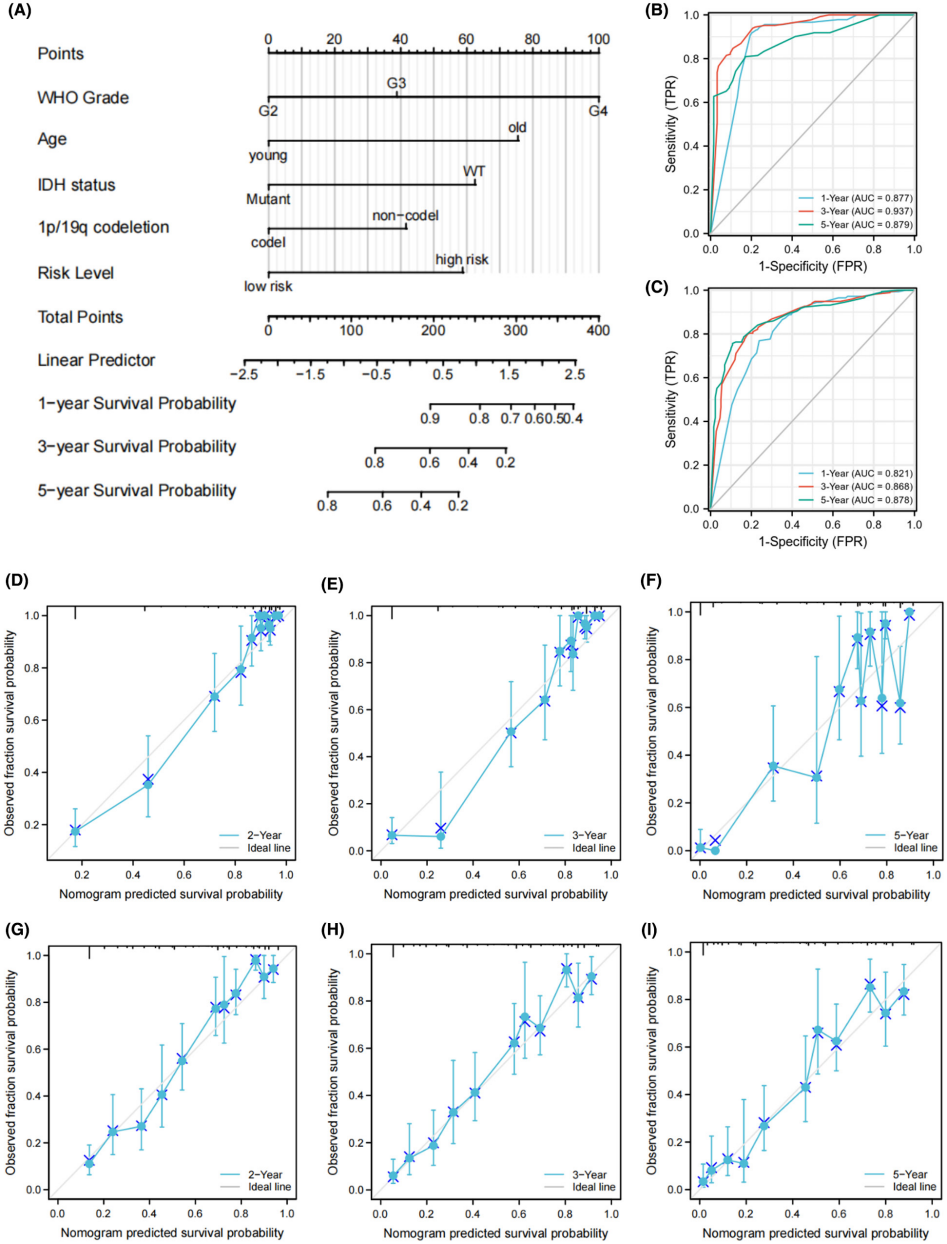
Construction and validation of the prognostic nomogram. (A) The nomogram was constructed by integrating independent risk factors screened by multivariate Cox regression. ROC curves of the nomogram in the (B) TCGA and (C) CGGA set. Calibration curves of the nomogram for predicting 2‐, 3‐, and 5‐year OS in the (D–F) TCGA sets and (G–I) CGGA sets.

To better evaluate the risk of glioma patients, a nomogram was constructed by combining the prognostic model and other independent risk factors. The C‐index of the nomogram model was 0.847 (95% CI = 0.837–0.858). As shown in Figure 3A, each item in the nomogram was assigned a point according to the actual condition of the case. The total points were calculated for each case and used to predict the survival rates. Calibration analysis and ROC curve analysis were performed to assess the prediction performance of the nomogram. In TCGA set, the AUC reached 0.877 at 1 year, 0.937 at 3 years, and 0.879 at 5 years OS rates of nomogram (Figure 3B). The calibration curves showed good fitting between the observed value and optimized value of 1‐, 3‐, and 5‐year OS rates (Figure 3D–F). In addition, the accuracy of our nomogram was also validated in the CGGA set. The AUC reached 0.821 at 1 year, 0.868 at 3 years and 0.878 at 5 years OS rates of nomogram (Figure 3C). Calibration curves also showed good fitting between the observed and optimized survival rate in CGGA set (Figure 3G–I). These results suggest that the nomogram has good predictive ability in both sets and can be used as a reference for clinical decision‐making.

### 3.5 GSEA

**FIGURE 4:**
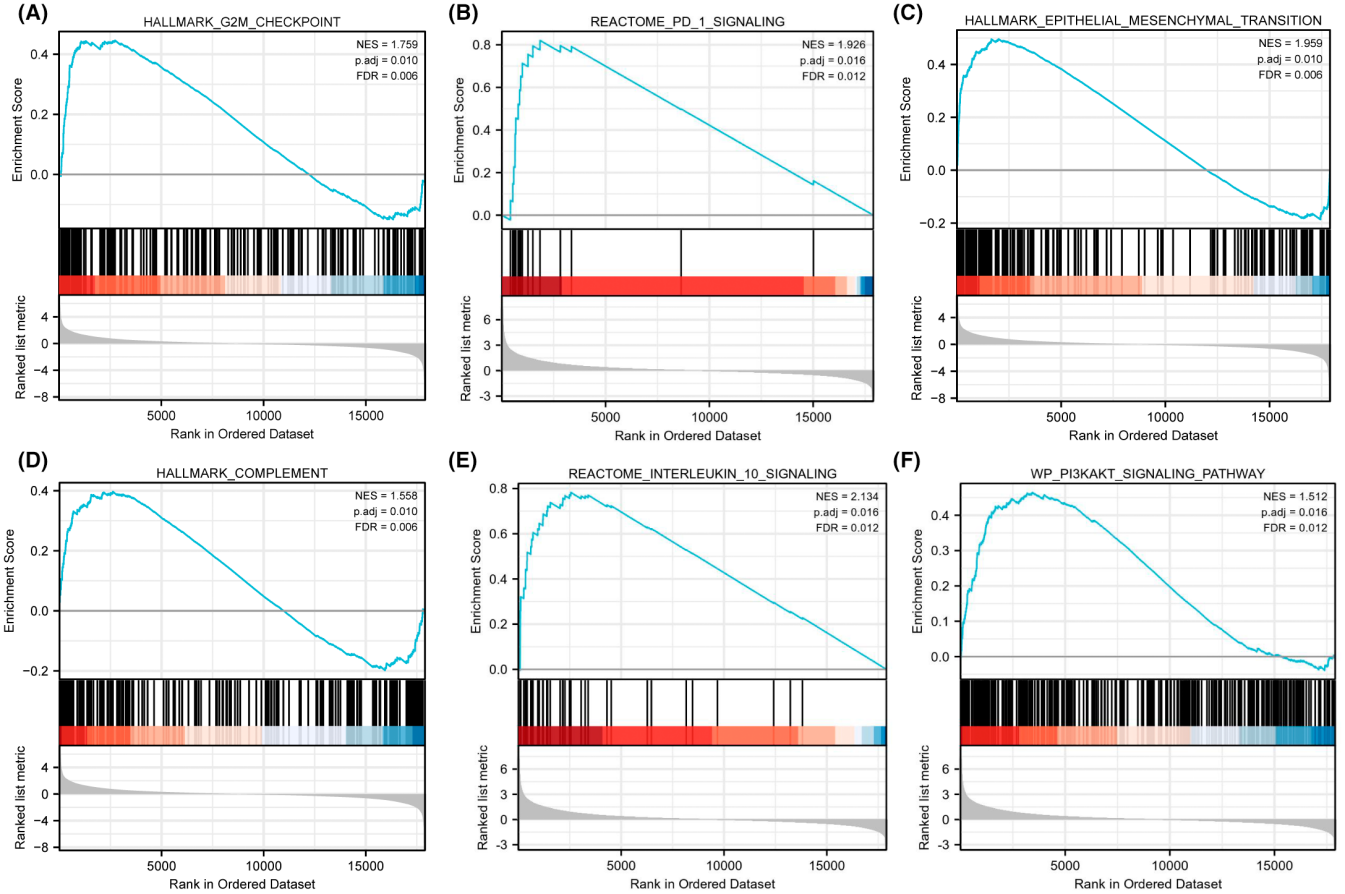
GSEA of the copper metabolism‐related gene signature. In the TCGA cohort, (A) G2M checkpoint, (B) PD‐1 signaling pathway, (C) epithelial–mesenchymal transition, (D) complement, (E) IL‐10 signaling, and (F) PI3K/AKT signaling were enriched in the high‐risk group.

According to the above analyses, patient prognosis differed significantly between two risk groups. To explore the reasons for this difference, we performed GSEA on genes expressed significantly differently between two risk groups in the TCGA set. GSEA results suggested that genes highly expressed in the high‐risk group were significantly enriched in G2M checkpoint, PD‐1 signaling, epithelial mesenchymal transition, complement, IL‐10 signaling, PI3KAKT signaling pathways (Figure 4A–F). As the PD‐1 signaling pathway and IL‐10 signaling pathway are key pathways for immunosuppression,^38^, ^39^ these results suggest that immunosuppression and innate immune dysregulation may play an important role in the progression of diffuse glioma, which further suggests that diffuse glioma is a kind of tumor that highly associated with immune dysfunction.

### 3.6 Immune infiltration analysis and mutation landscape

**FIGURE 5:**
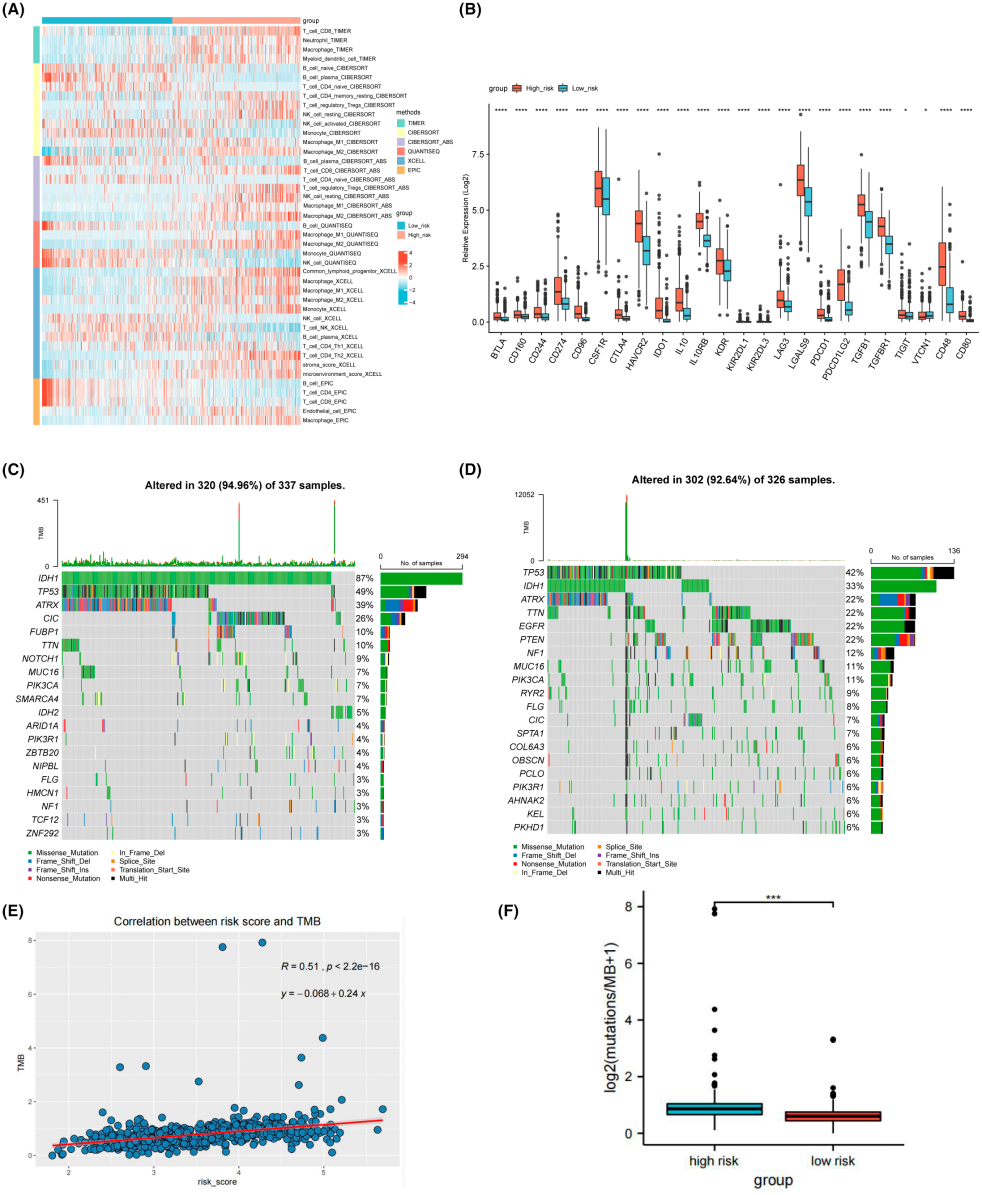
Immune infiltration analysis and mutation landscape in TCGA dataset. (A) Heatmap of immune cell infiltration in the high risk group and low risk group. (B) Comparison of the expression of immune checkpoint‐related genes in the high‐risk group and low‐risk group. (C) Mutation landscape of the low‐risk group. (D) Mutation landscape of the high‐risk group. (E) Barplot of TMB in the low‐risk group and high‐risk group, compared by Mann–Whitney *U*‐test. (F) Correlation between TMB and risk scores. *p < 0.05; **p < 0.01; ***p < 0.001; ****P < 0.0001.

Based on the results of GSEA, the immunosuppressive tumor microenvironment (TME) may play a role in the progression of diffuse glioma, so the comparison of immune infiltration levels between two groups in the TCGA dataset was performed. As shown in Figure 5A, in the high‐risk group, there were more immune cells infiltration, including CD8+ T cells, regulatory T cells, neutrophils, macrophages, myeloid dendritic cells, and higher stroma in tumor tissue. Next, we explored the expression of immune checkpoint genes. As shown in Figure 5B, the expression levels of most immune checkpoint genes, such as CD274, PDCD1, LAG3, CTLA4, and HAVCR2, are higher in the high‐risk groups (*p* < 0.0001). The high expression of immune checkpoint genes may result in suppressed immune cell function and blunted immune response. Therefore, although the high‐risk group was related to a higher level of immune cell infiltration, the tumor cells remodel the TME by regulating the immune checkpoint genes expression of tumor cells and immune cells, thus inhibit antitumor immunity and lead to tumor immune evasion, which can be one of the explainations for the different prognosis between two risk groups.

To further explore the potential role of the prognostic model in predicting the response of immunotherapy, we analyzed the mutation landscape in the TCGA dataset. In the low‐risk group, the mutation incidence was 94.96% and the most significantly mutated gene was IDH1 (Figure 5C). While the mutation incidence in the high‐risk group was 92.64%, and the most significantly mutated gene was TP53 (Figure 5D). Figure S3 shows the overall mutated genes with significantly different mutation incidence between the two groups. Furthermore, as TMB was found to be a predictive biomarker for the clinical efficacy to immunotherapy,^40^ we calculated the correlation between risk score and TMB for patients in the TCGA‐LGG/GBM samples. The result showed that TMB was positively correlated with risk score (*R* = 0.51, *p* < 0.0001, Figure 5E). In addition, the TMB was significantly higher in the high‐risk group than in the low‐risk group (*p* < 0.001; Figure 5F). Therefore, these results indicated that patients in the high‐risk group may benefit more from immunotherapy.

### 3.7 Aberrant expression of the identified genes in glioma samples detected by IHC and survival analysis

Subsequently, we verified the protein expression level of these identified genes by immunohistochemical analysis. First, we searched these genes in HPA database. Among them, LOXL3 and FDX1 were not detected in LGG or GBM samples. NFE2L2 protein highly expressed in LGG while moderately expressed in GBM. CDK1 and LOXL2 had low expression in GBM while were not detected in LGG (Figure S4).

**FIGURE 6:**
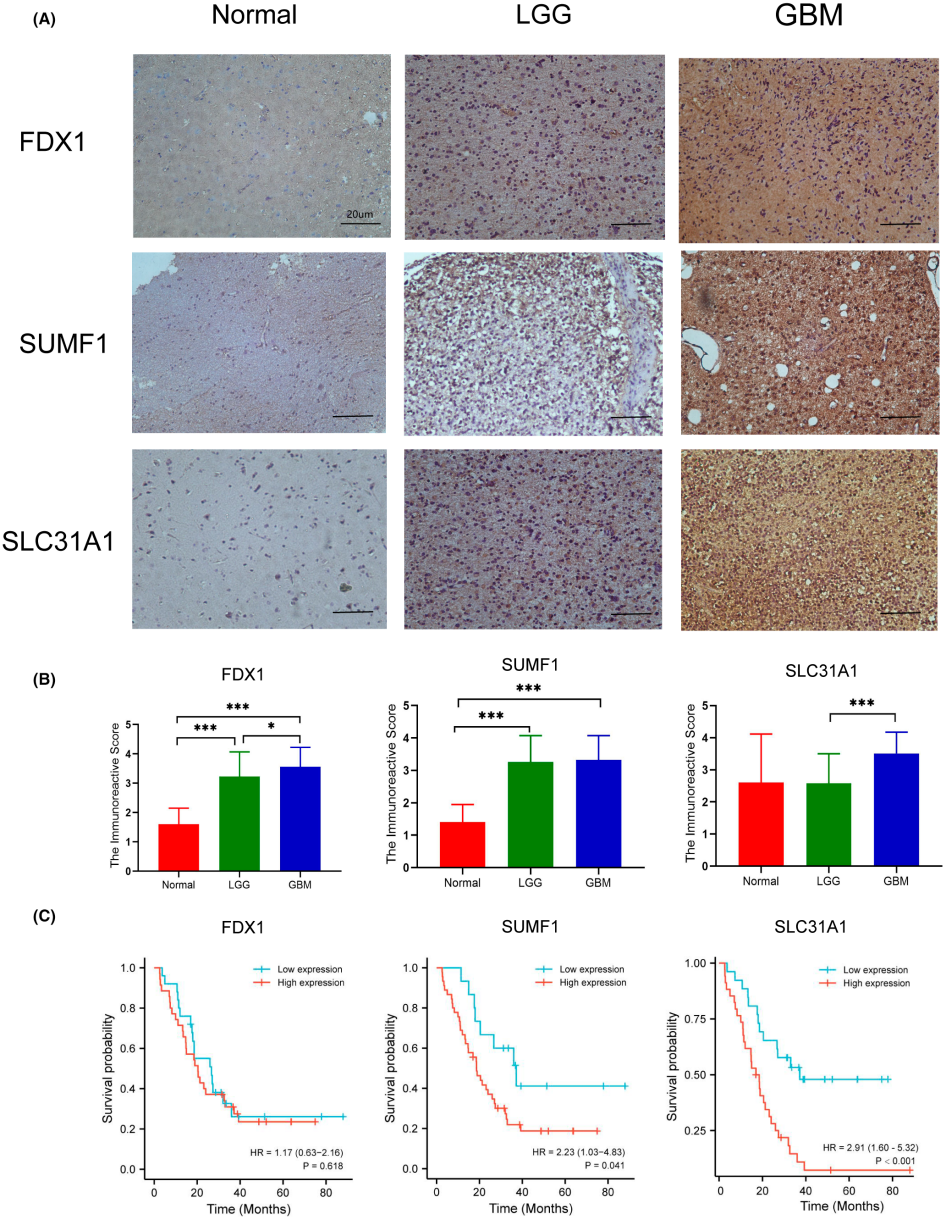
FDX1, SUMF1, and SLC31A1 protein expression in normal brain tissue, LGG and GBM samples using immunohistochemical analysis. Normal control was collected from patients with benign cerebral diseases. (A) Representative images of immunohistological analysis. Scale Bar = 20 μm (B) Summary of FDX1, SUMF1, and SLC31A1 protein expression profile (Mann–Whitney *U*‐test). *p < 0.05; **p < 0.01; ***p < 0.001. (C) Kaplan–Meier survival analyses were performed to compare the OS times between the high expression and low expression of FDX1, SUMF1, and SLC31A1 protein.

Since the HR values of FDX1, SUMF1, SLC31A1 were the highest in the seven‐gene signature, we were going to validate their protein expression in human glioma specimens. The aberrant protein expressions of these three genes in glioma samples were confirmed by IHC (Figure 6A,B). FDX1 shows higher protein expression in GBM and LGG relative to normal brain tissue (Both *p* < 0.001). Notably, the expression of FDX1 protein in GBM is significantly higher than that in LGG (*p* < 0.05). The expression of SLC31A1 protein in GBM was significantly higher than that in LGG (*p* < 0.001). The expression of SUMF1 protein in GBM and LGG was significantly higher than that in normal brain tissue (Both *p* < 0.001).

As for survival analysis, the high expression of FDX1 and SLC31A1 protein was associated with poor survival in glioma patients (*p* < 0.05 for FDX1 and *p* < 0.001 for SLC31A1, Figure 6C). However, there was no significant correlation between the protein expression of SUMF1 and OS time in patients with gliomas.

## 4 DISCUSSION

Gliomas are highly heterogeneous and refractory tumors with high recurrence rates and resistance to chemo‐ and radiotherapy.^41^ Copper is an important nutrient involved in many biological functions; however, due to its redox properties, abnormal copper levels can be toxic to cells. The connection between copper and copper‐dependent diseases, especially cancer, has been noted, since numerous observations have shown that tumors have a greater demand for copper compared to healthy tissue. Furthermore, elevated copper levels have been proven to be directly related to cancer progression.^42^ Copper metabolism was reported to take part in glioma proliferation, angiogenesis,^43^ and the TME.^26^ Copper metabolism‐related genes are abnormally expressed in tumor tissues relative to normal tissues,^44^ and thus emerge as potential prognostic markers for gliomas. However, there are still insufficient studies in exploring the relationship between copper metabolism and clinical characteristics among glioma patients.

In the current study, we conducted a bioinformatic analysis using gene expression and survival data of from TCGA, and screened for 16 DEGs with prognostic value. Then, seven genes were screened to construct a prognostic model by LASSO regression analysis. The prognostic model consisted of seven genes: CDK1, LOXL2, LOXL3, NFE2L2, SLC31A1, SUMF1, and FDX1.

CDK1, a kinase related to the cell cycle, was found to be up‐regulated by temozolomide (TMZ) in a NF‐κB dependent way in GBM,^45^ and can be a target for glioma treatment by affecting the cell cycle.^46^ LOXL2 and LOXL3, members of the lysyl oxidase (LOX) family, LOXL2 was found to promote glioma progression, and improve the TMZ resistance of glioma cells.^47^ A study found that downregulation of LOXL3 decreased cell proliferation and invasion, and increased cell adhesion in U87 cells.^48^ NFE2L2 is a transcription factor which protects cells from the oxidative damage and is associated with resistance to cancer treatments,^49^ which was indicated as a prognostic indicator in gliomas.^50^ SLC31A1 encodes copper transporter 1 (CTR‐1), which is a transmembrane pump responsible for copper absorption. It is highly expressed in different types of cancer characterized by elevated copper levels, and high CTR‐1 expression enhances PD‐L1 expression by promoting cellular copper influx, aggravating PD‐L1‐driven tumor immune escape.^51^ SUMF1, has been reported whose increased expression could improve relapse‐free survival of breast cancer patients.^52^ FDX1, which encodes a reductase that reduce Cu^2+^ to its more toxic form, Cu^1+^, is a direct target of elesclomol (a copper‐loaded ionophore).^53^ Peter Tsvetkov found through knockout studies that FDX1 deletion confers resistance to copper‐induced cell death. Since cells with high levels of FDX1 are sensitive to copper‐induced cell death, the authors suggest that copper ionophore treatment should be targeted at tumors with this metabolic profile.

Based on GSEA, genes highly expressed in the high‐risk group were enriched in pathways related to tumor progression and immune response. It is reasonable to assume that copper metabolism may be closely linked to tumor progression and TME in glioma. Kenichi reported that copper ionophore NSC319726 induced DNA damage, causing cell cycle arrest in glioblastoma cells. However, it showed no substantial toxicity to differentiated and ungrown neurons, and this selective lethality of copper ionophore is an ideal feature as an anti‐GBM drug.^54^ PD‐1 is an immunosuppressive receptor that causes T‐cell dysfunction and apoptosis by binding to its ligand PD‐L1^55^ and plays a role in braking inflammatory responses and conspiring immune evasion of glioblastoma cells.^56^ Epithelial–mesenchymal transition (EMT) is the main cause of recurrence and poor prognosis of glioma.^57^ IL‐10 is an anti‐inflammatory cytokine produced mainly by M2 macrophages.^58^ Vidhya M found that IL‐10 release drives T‐cell exhaustion in glioblastoma, thereby contributing to an immunosuppressive TME.^59^ The PI3K/Akt/mTOR signaling pathway is one of the key pathways in tumor progression, and its molecular alteration is a typical hallmark of glioma.^60^, ^61^

We further explored the heterogeneity of the tumor immune environment between two groups to look for potential causes of different outcomes. The risk score was positively correlated with the level of immune cell infiltration and stromal infiltration, which was consistent with previous study indicating that tumor‐surrounding stromal cells, such as tumor‐associated macrophages (TAMs), myeloid‐derived suppressor cells (MDSCs), and regulatory T cells (Tregs), could produce chemokines to promote tumor growth, resulting in poor prognosis in patients.^62^ In addition, the higher expression levels of immune checkpoint proteins in patients in the high‐risk group, where the more immunosuppressive TME predominated, provides a plausible explanation for their poorer survival outcome.

TMB is an emerging potential biomarker for immunotherapy.^63^ A prevailing view in the field of immunotherapy is that tumors with increased TMB present more neoantigens and therefore are correlated with better clinical response to immunotherapy.^64^ One of the major obstacles to effective immunotherapy for GBM is the low TMB in most GBM tumors.^65^ Our study showed a positive correlation between risk scores and TMB in gliomas, which may indicate that the patient with higher risk score respond better to immunotherapy.

As cuproptosis was identified to be a novel mechanism of cell death, the role of copper metabolism in tumor progression and its guiding value for treatment have attracted much attention. Some studies have proved that copper ionophores may contribute to the treatment of glioma,^54^ but no study has yet explored the value of the copper metabolism‐related genes in predicting the prognosis and guiding the treatment of glioma. In this study, copper metabolism‐related gene prognostic model for glioma was developed, and the clinical features of glioma patients were further integrated to develop a nomogram to better predict the survival of glioma patients. Compared with other published models, our model has better prediction efficiency (Table S2). In addition, we explored the potential mechanisms of different outcomes of glioma patients from the perspective of tumor immune microenvironment, and predicted the possible immunotherapy response of glioma patients. We further confirmed the abnormal expression of FDX1, SUMF1, and SLC31A1 proteins in glioma samples by immunohistochemistry, and the correlation between high expression of FDX1 protein and poor prognosis in glioma patients. Considering that FDX1 is a key regulator mediating cuproptosis, our study also provided a new perspective and direction for the future research on the treatment of glioma using the cuproptosis mechanism.

However, this study also has some limitations. First, immune microenvironment analysis and mutation analysis were not validated in the testing set due to lack of data. Second, in this study, only partial genes were verified for protein expression and survival analysis in clinical samples, without further experiments in vitro or in vivo to explore their roles and mechanisms in glioma.

## 5 CONCLUSION

In conclusion, we constructed a copper metabolism‐related gene prognostic model in glioma for the first time. Using this model, we can well predict the prognosis and the possible immunotherapy response of glioma patients. We further confirmed the abnormal expression of FDX1, SUMF1, and SLC31A1, and found the correlation between high expression of FDX1 and SLC31A1 protein and poor prognosis in glioma patients, which provided a new direction for the future research on the treatment of glioma using the cuproptosis mechanism.

## AUTHOR CONTRIBUTIONS

**Ling Li:** Conceptualization (lead); data curation (lead); formal analysis (lead). **Wenyuan Leng:** Conceptualization (supporting); data curation (supporting); formal analysis (supporting); writing – original draft (supporting). **Junying Chen:** Data curation (supporting); formal analysis (supporting); writing – original draft (supporting); writing – review and editing (supporting). **Shaoying Li:** Validation (lead). **Bingxi Lei:** Resources (lead). **Huasong Zhang:** Project administration (lead); writing – review and editing (equal). **Huiying Zhao:** Funding acquisition (lead); project administration (supporting); writing – review and editing (equal).

## FUNDING INFORMATION

The work was funded by the National Key Research and Development Program of China (2020YFB0204803), the Natural Science Foundation of China (81801132 and 81971190), Guangdong Key Field Research and Development Plan (2019B020228001 and 2018B010109006), and Natural Science Foundation of Guangzhou (2021A1515010256). Medical and Health Science and Technology Plan of Longgang Shenzhen (No. LGKCYLWS2022019).

## CONFLICT OF INTEREST STATEMENT

The authors declare no conflict of interest.

## DISCLOSURE

None.

## ETHICS STATEMENT

All samples were collected with informed consent according to the Internal Review and the Ethics Boards of the Sun Yat‐sen Memorial Hospital of Sun Yat‐sen University (SYSKY‐2023‐108‐01).

## Supporting information


Figure S1.

Figure S2.

Figure S3.

Figure S4.

Table S1.

Table S2.

Appendix S1.

## Data Availability

Data sharing is not applicable to this article as no new data were created or analyzed in this study.
