# Supplementary figures and images for: Identification of a copper metabolism‐related gene signature for predicting prognosis and immune response in glioma
Source: Cancer Med. 2023 Mar 1;12(8):10123–37. doi: 10.1002/cam4.5688 (PMC10166918; doi:10.1002/cam4.5688)

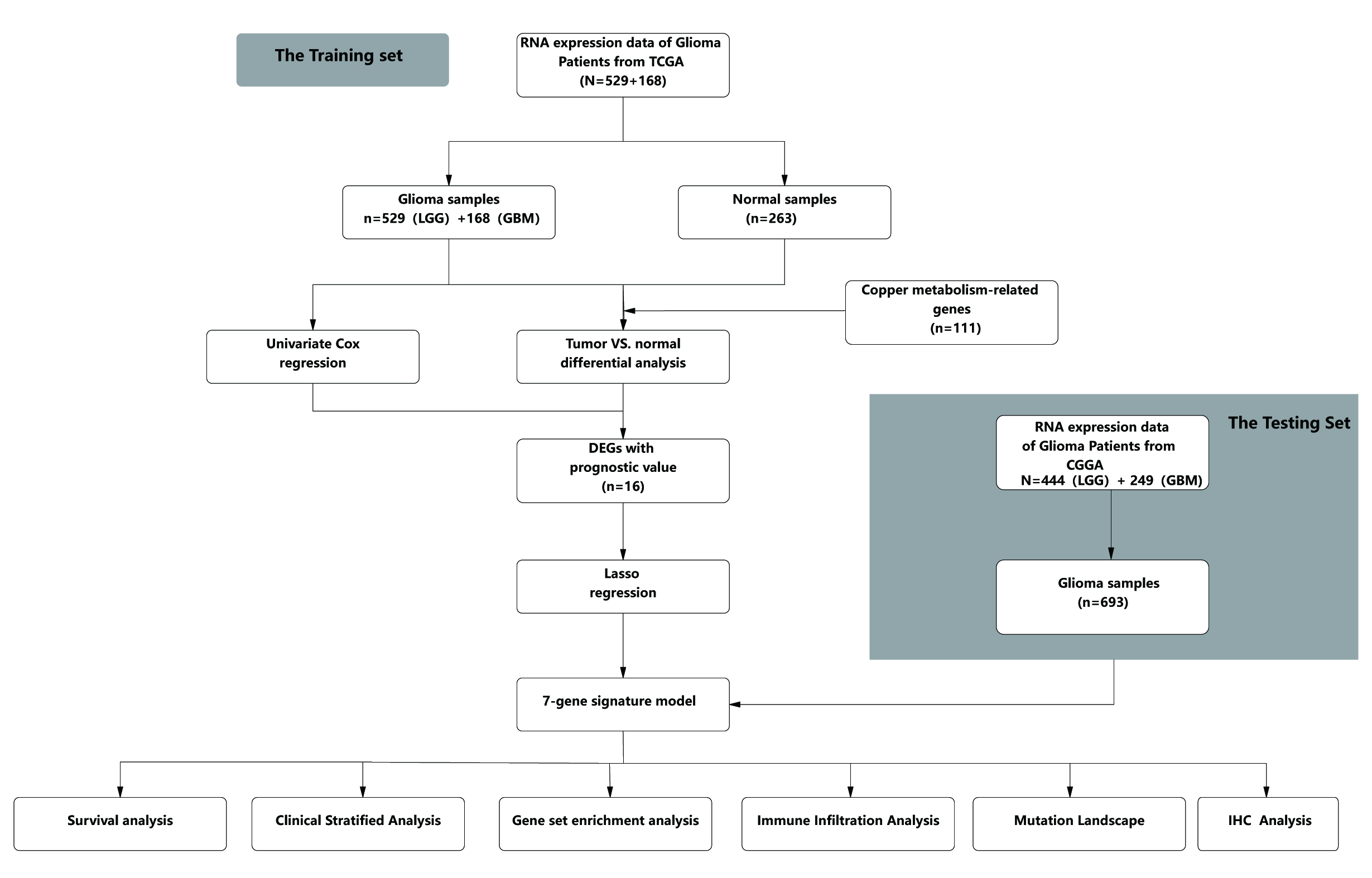

Supplement: Supplementary file 1 — Figure S1. [file CAM4-12-10123-s004.tif]

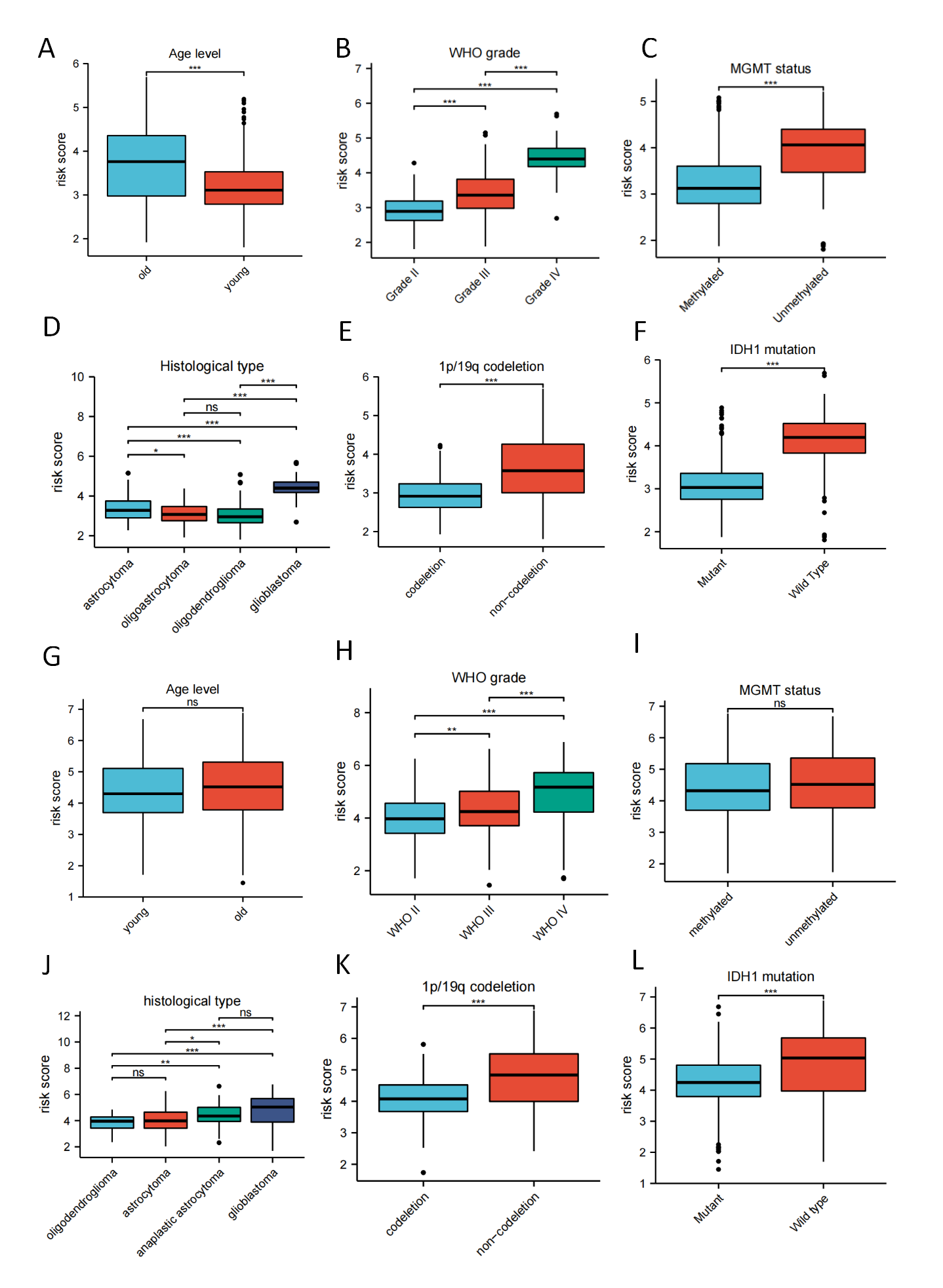

Supplement: Supplementary file 2 — Figure S2. [file CAM4-12-10123-s005.tif]

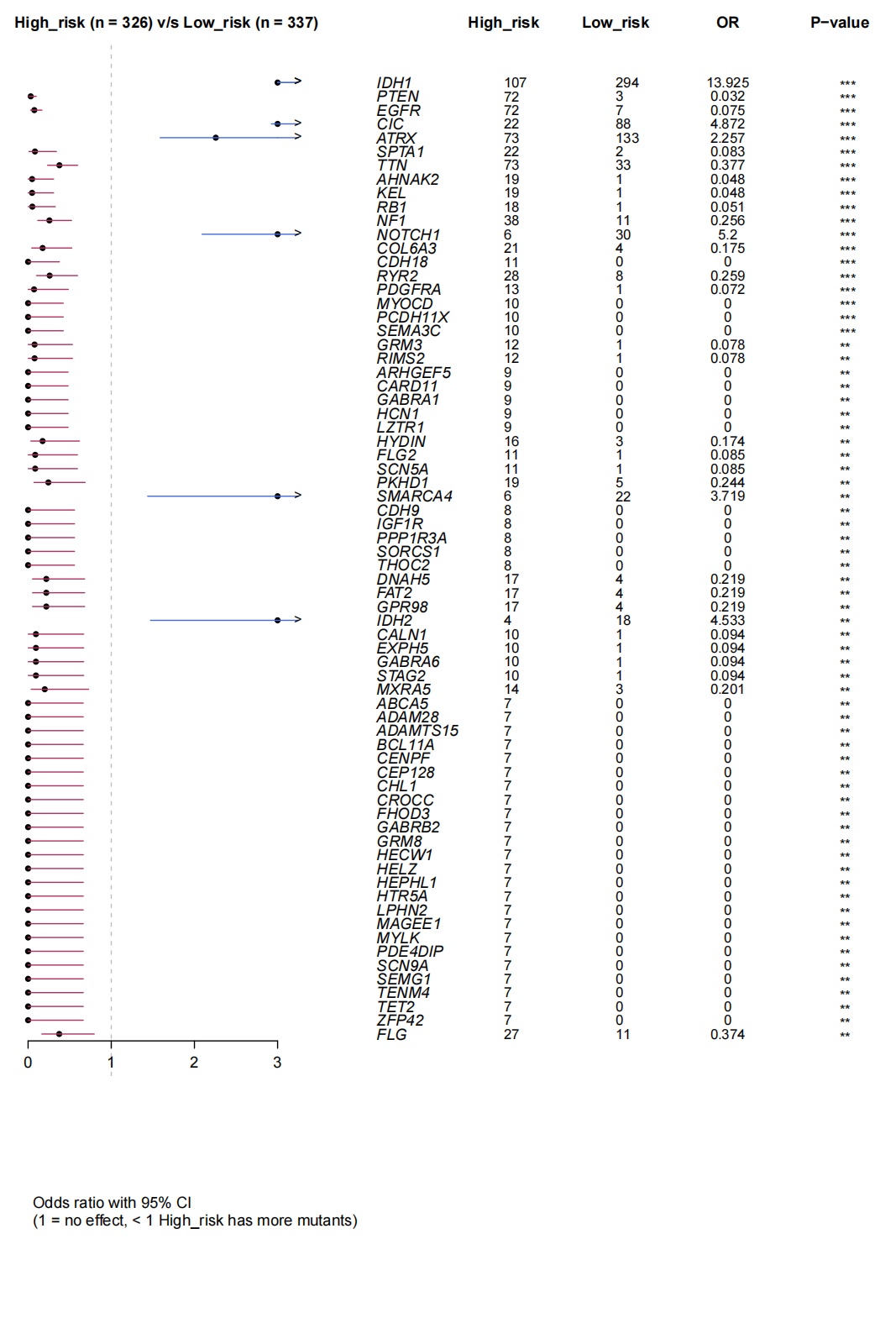

Supplement: Supplementary file 3 — Figure S3. [file CAM4-12-10123-s006.tif]

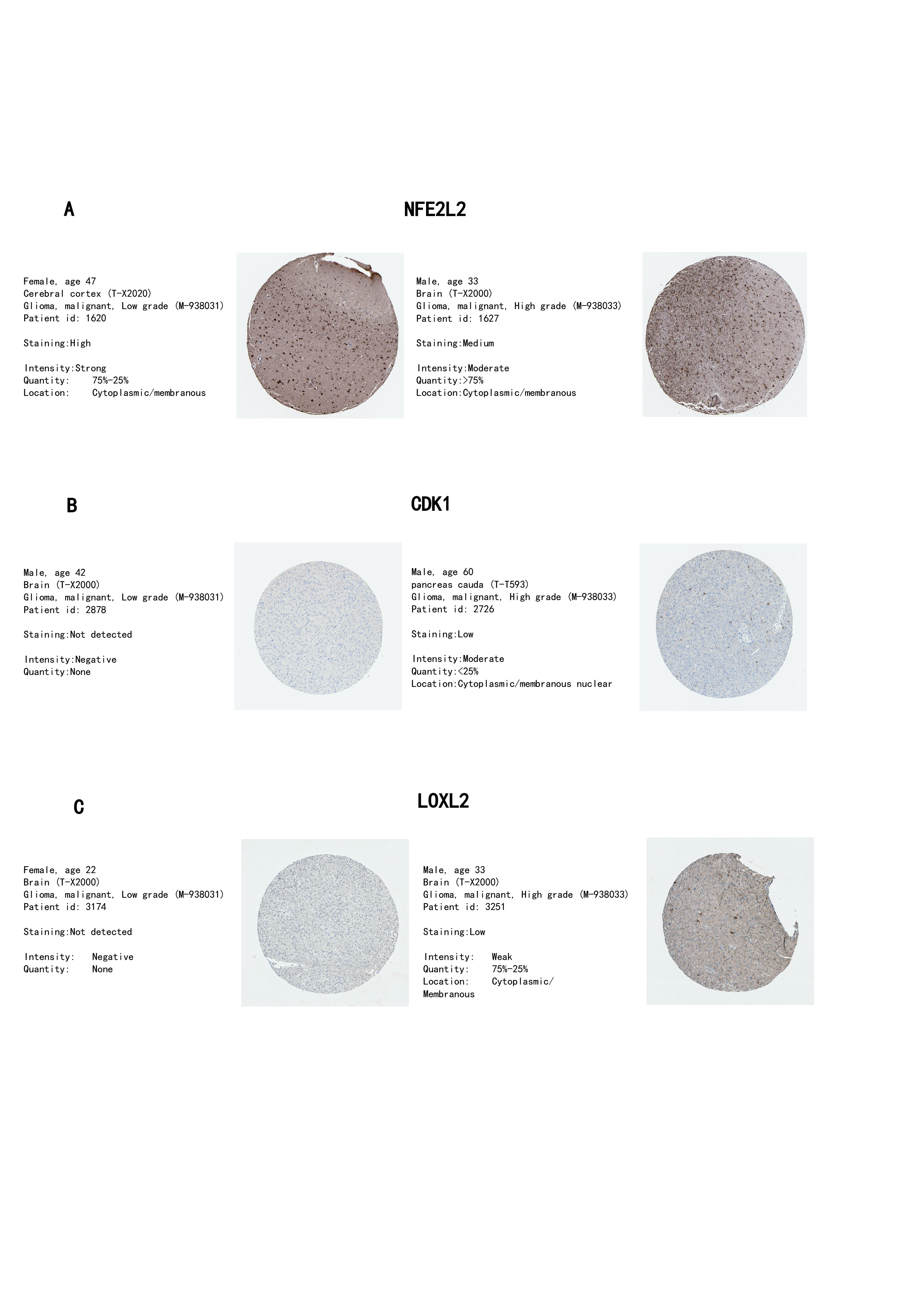

Supplement: Supplementary file 4 — Figure S4. [file CAM4-12-10123-s003.tif]
