# Supplementary material for: Identification of a copper metabolism‐related gene signature for predicting prognosis and immune response in glioma
Source: Cancer Med. 2023 Mar 1;12(8):10123–37. doi: 10.1002/cam4.5688 (PMC10166918; doi:10.1002/cam4.5688)
Supplement: Supplementary file 6 — Table S2. [file CAM4-12-10123-s001.doc]

| **Model** | **AUCs of Training set** | **AUCs of Testing set** | **C-index** | **Validation**  **Experiment** | **Method** |
| --- | --- | --- | --- | --- | --- |
| Our Model | 0.877 for 1 year,  0.937 for 3 years,  0.879 for 5 years. | 0.821 for 1 year,  0.868 for 3 years,  0.878 for 5 years | 0.847 | IHC | LASSO |
| Wang, W., et al.^[1]^ | 0.898 for 1 year,  0.918 for 3 years,  0.828 for 5 years. | Data not  provided | Data not provided | IHC and Western blot | Random Survival Forest model |
| Chen, B., et al.^[2]^ | 0.756 | 0.617 | Data not provided | Not done | LASSO |
| Zhu, H., et al.^[3]^ | Data not  provided | Data not  provided | 0.834 | Not done | LASSO |
| Zhang, Y., et al.^[4]^ | 0.773 for 1 year,  0.766 for 2 years,  0.784 for 3 years. | 0.692 for 1 year,  0.756 for 2 years,  0.754 for 3 years. | Data not provided | Not done | LASSO |

TABLE S2 | Comparison between our model and other published models.

|  |
| --- |
|  |

**References:**

1.Wang W, Lu Z, Wang M, Liu Z, Wu B, Yang C, Huan H, Gong P. Front Immunol(Frontiers in immunology), 2022, 13:998236.

2.Chen B, Zhou X, Yang L, Zhou H, Meng M, Zhang L, Li J. Comput Biol Med(Computers in biology and medicine), 2022, 148:105924.

3.Zhu H, Wan Q, Tan J, Ouyang H, Pan X, Li M, Zhao Y. Front Genet(Frontiers in genetics), 2022, 13:992995.

4.Zhang Y, Dai X, Li Z. Am J Transl Res(American journal of translational research), 2022, 14(11):8085-8102.
