# Supplementary material for: Identification of a copper metabolism‐related gene signature for predicting prognosis and immune response in glioma
Source: Cancer Med. 2023 Mar 1;12(8):10123–37. doi: 10.1002/cam4.5688 (PMC10166918; doi:10.1002/cam4.5688)
Supplement: Supplementary file 7 — Appendix S1. [file CAM4-12-10123-s007.docx]

**Supplementary Methods**

**Building and Validating the Prognostic Model**

According to the TCGA set, the copper metabolism-related DEGs were combined into a univariate Cox analysis of overall survival (OS) using “survival” package, and the genes with Cox P value < 0.05 were considered to be copper metabolism-related genes with prognostic values. Functional enrichment analysis of the prognostic copper metabolism-related genes was performed using Metascape. Then, LASSO regression analysis was applied to construct a prognostic model, which has reduced the overfitting by selecting high-dimensional prognostic genes using the “glmnet” package. The risk score was calculated using the following formula:

$Risk Score = \sum_{i=1}^{n} {expr}_{genei}\times\mathrm{coefficient}_{genei}$

Where ${expr}_{genei}$ is the normalized gene expression value and $\mathrm{coefficient}_{genei}$ is its coefficient.

Through this formula, each LGG/GBM patient can be calculated with a risk score, and patients were grouped into high-risk or low-risk group according to the median risk score. Then, the risk score was used to predict survival of patients. The “timeROC” package was used to conduct the time-dependent receiver operating characteristic (ROC) curve to evaluate the predictive performance. Besides, the CGGA dataset was set as a testing set to validate the predictive performance of the risk score.

**The Construction of a Nomogram**

To better predict the prognosis in both the training and testing cohorts, a nomogram was constructed. Firstly, the risk level and potential risk factors, including gender, WHO grade, age, IDH1 mutation status, 1p19q co-deletion status, and MGMT promoter methylation status, were used as parameters in univariate Cox regression analyses to find risk factors for OS time. Parameters of Cox P value <0.05 in univariate Cox regression were further analyzed by multivariate Cox regression to assess the independence of the risk factors. Parameters of P value <0.05 in multivariate Cox regression analysis were then integrated to construct a nomogram by the “rms” package. Calibration analysis and ROC curve were used to assess the accuracy of the nomogram.

**Mutation Landscape**

Mutational data of patients (n=681) with LGG/GBM from TCGA were obtained from cBioPortal (http://www.cbioportal.org). The mutational data was analyzed by “maftools” R package. We explored the differences in mutations between high-risk and low-risk groups and displayed the top 20 genes with the highest mutation frequency. Besides, we identify the association between risk score and TMB in patients with LGG/GBM by Spearman’s correlation test.

**Tissue Specimens and Patient Information**

The median follow-up time of these 60 patients, including 26 females and 34 males (age range, 25–72 years; median, 48 years), was 20.4 months, and the maximum follow-up time was 88 months. None of the patients was treated with either chemotherapy or radiation prior to surgery. The study was approved by the ethics committee of Sun Yat-sen University , and all samples were collected with informed consent from all subjects.

**Immunohistochemical (IHC) staining**

For immunohistochemical (IHC) staining, according to the instructions of antibody manufacturers, tissue slides or TMAs of human surgical specimens were deparaffinized, rehydrated through an ethanol series, followed by antigen retrieval with sodium citrate solution. The sections were then incubated with 1% Triton-X100 for 15 min, 3% H_2_O_2_ for 10 min, blocked with 5% normal goat serum for 30 min at room temperature, and then incubated with appropriate primary antibodies at 4 ℃ overnight. Primary antibodies used include rabbit anti-FDX1 (1:100, ABclonal A20895), rabbit anti-SUMF1 (1:100, Bioss, bs-12366R) and mouse anti-SLC31A1 (1:100, Proteintech, 67221-1-Ig). IHC staining was performed with horseradish peroxidase (HRP) conjugates using DAB detection. The immunoreactive score (IRS) was calculated as the percentage of positive cells (A): 0 = no positive cells; 0.3 = 30% positive cells, 0.5 = 50% positive cells, 0.8 = 80% positive cells, 1 = 100% positive cells. The IRS also calculated for intensity of staining (B): 1 = no color reaction; 2 = mild reaction; 3 = moderate reaction; 4 = intense reaction. The immunoreactive score (IRS Score) = A×B.

**Statistical Analysis**

GraphPad Prism 8.0 was used for statistical analysis. Kaplan–Meier survival data were analyzed using two-sided log-rank test. The two-group comparisons were analyzed by unpaired two-sided student's t test and the Mann-Whitney U test. P values <0.05 were considered significant.
